# Surgical Treatment of Post-Traumatic Radio-Ulnar Synostosis

**DOI:** 10.3390/medicina60122026

**Published:** 2024-12-09

**Authors:** Mihai Tudor Gavrilă, Vlad Cristea, Cătălin Gabriel Smarandache, Cristea Ștefan

**Affiliations:** 1 Department of Orthopedics and Traumatology, St Pantelimon Emergency Hospital, 021659 Bucharest, Romania; drstefancristea@yahoo.com; 2 Department of Orthopedics and Traumatology, Colentina Hospital, 020125 Bucharest, Romania; vladcristea@hotmail.com; 3 Department of General Surgery, Bucharest Emergency University Hospital, 050098 Bucharest, Romania

**Keywords:** radio-ulnar synostosis, stiffness, surgery, resection, recurrence, AINS, radiotherapy

## Abstract

Radio-ulnar synostosis is a rare complication which develops following forearm trauma, the main manifestation being stiffness and leading to the loss of pronation and supination. For the patient, it is a very frustrating experience due to the impairment of the normal function of the forearm, whereas for the surgeon the treatment is difficult as, unfortunately, there is no consensus regarding the best way to approach it. Many surgical techniques and other kinds of adjuvant therapies have been developed in an effort to solve this disability. This paper presents an overview of the principal factors which contribute to the development of synostosis and the best therapeutic approach methods found in the literature.

## 1. Introduction

The radius and the ulna are articulated in two places, proximally and distally, creating two pivot joints that enable the pronation and supination of the forearm. Traumatisms at the level of the forearm bones (such as fractures) create circumstances to develop radio-ulnar synostosis, which is a fibrous or osseous fusion, similar to a bridge, between both bones, having a severe functional impact on professional and everyday life as the patient loses pronation and supination movements and sometimes even develops stiffness in the elbow joint. The incidence of synostosis varies from 0% to 9,4%, depending on the severity of the trauma of the forearm.

Normal pronation and supination movement is 80–90 degrees for each one, starting from the neutral point. In radio-ulnar synostosis, the degree of limitations varies from a mild form to the complete absence of pronation and supination, the forearm remaining stiff in neutral position. When the synostosis is situated proximally at the level of the forearm, it can include the elbow joint, producing a stiff elbow. Because of this possible dysfunction, it is important to know the factors that can lead to the formation of synostosis. There are some risk factors, but prevention is possible only for a few of them. All of them will be detailed further in the paper. The main treatment for synostosis is surgical and consists of the resection of the fusion, with or without interposition of soft tissue. To prevent recurrences, some authors recommend different adjuvant treatments with variable results.

## 2. Methodology

To provide an overview of the pathology and the main treatment methods, we analyzed the most relevant publications from the last 50 years. The working strategy was to systematically present the main classifications that guide surgical interventions, followed by the presentation of several series of cases with associated adjuvant treatments.

Although the variety of options offered may seem confusing, the goal is to provide the broadest and most detailed information as possible to allow for practicing surgeons to adopt the best strategy in the treatment of radio-ulnar synostosis.

## 3. Epidemiology and Physiopathology

**Figure 1:**
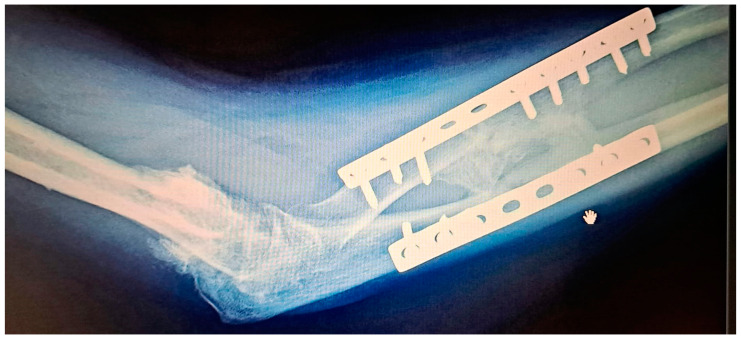
Radio-ulnar synostosis after a car accident involving the elbow and forearm.

Bone fusion can occur anywhere along the forearm (from the wrist to the elbow) and is a rare complication of the traumatisms of the radius and ulna (Figure 1). Depending on the case series examined, the incidence is from 0 to 9,4% of forearm fractures [1,2,3,4]. Every traumatism of the interosseous membrane can lead, in some circumstances, to the formation of fibrous tissue or a bone bridge (from hardware such as screws, external fixators, or plates used in surgery) [5,6]. Because the radius has a normal movement of rotation over the ulna in pronation, the new bone bridge formation (synostosis) between these two bones blocks it. Extension of the bone proximal to elbow provokes stiffness of the joint.

Not all traumatic events produce synostosis. There are some risk factors involved, such as car accidents with a high level of soft tissue injury, comminuted fractures with fragments in the interosseous space, fractures of the forearm, of both bones at the same level, fracture-luxation (Monteggia fracture), brain injuries, surgical delay, a single approach for both bone fractures, inappropriate length of screws (too long), and prolonged immobilization with late rehabilitation [5,7,8].

### 3.1. Classification

**Figure 2:**
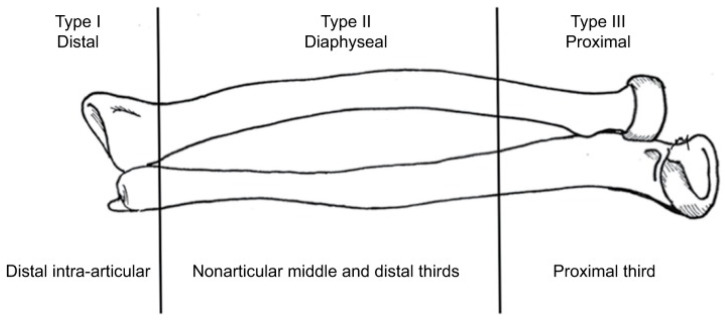
Vince and Miller radio-ulnar synostosis classification.

In 1987, Vince and Miller proposed an anatomical classification based on the location of the synostosis along the bones of the forearm [2] (Figure 2).

Type I synostosis is situated in the distal intra-articular portion of the radius and the ulna. In the Vince series, it was associated with poor prognosis (100% failure).

Type II synostosis is localized in the middle third of the forearm (is secondary to severe trauma and had the best results).

Type III synostosis is localized in the proximal third of the forearm (the rate of failure was also high in the Vince series).

Subsequently, Jupiter and Ring modified this classification by sub-classifying Type III into Type III A (distal, or at the level of the bicipital tuberosity), Type III B (at the level of the radial head), and Type III C (when heterotopic ossification continues from the elbow or distal humerus) [4].

To offer help for the surgical treatment, Hastings and Graham modified the Vince classification in their own way [9].

### 3.2. Clinical Manifestations

In complete radio-ulnar ossification, the forearm is pain-free, but pronation and supination are completely absent in both passive and active mobilization. Sometimes, there can be a few degrees of movement in the distal radio-carpal joint. In the early stage of pre-ossification (the fibrous stage), there can be a little pain associated with limited pronation and supination. If the ossification extends from the elbow, or from the distal humerus to the radius or the ulna, the elbow is blocked.

### 3.3. Imaging Investigations

Usually, an X-ray investigation is mandatory in order to make the diagnosis. Plain AP and lateral (sometimes three-quarter) radiographs allow the physician to identify and localize the synostosis. Furthermore, a CT scan helps the surgeon to evaluate the extension and dimensions of the osseous bridge. This helps in surgical planning. Some authors recommend a bone scan to evaluate the maturation of the osseous tissue [9]. In paraplegic patients, a high level of alkaline phosphate was found in the blood, especially during the formation of the ossification. Once the new bone was mature, these levels normalized [10]. It is difficult to say if this investigation is available for patients with post-traumatic radio-ulnar synostosis.

### 3.4. Treatment

The treatment of radio-ulnar synostosis is surgical. Exceptions to this rule are patients with low-demand forearm function, those with severe associated comorbidities, patients who do not accept the risk of a surgical intervention, or patients with such extensive synostosis that total resection appears impossible. A conservative treatment also works for patients with an acceptable radio-ulnar arc of motion. For the rest of the patients, ossification can only be removed through an open operation, thus restoring the radio-ulnar movement. The purpose of the surgical intervention is to restore forearm rotation.

The timing of the surgical intervention is controversial. If is too early, there is a risk of recurrence; if is too late, there is a risk of peri-articular soft tissue retraction and the surgery is inefficient. The guidelines in use are based on radiographic investigations (Hasting and Graham classification), bone scans, and serum alkaline phosphate.

Some authors report good results before 6 months [11,12] with the early restoration of joint movement, but the general consensus is to operate on synostosis between 6 months and 2 years after the injury, with the best results gained between 1 and 2 years [2,8,13,14].

Jupiter and Ring reported no significant difference in pronation and supination between patients (a series of 18) operated on during the first months after injury (with adjuvant postoperative radiation therapy) [4].

McAulife and Wolfson [15] specify that none of the eight patients operated on for ossification (with postoperative adjuvant radiation therapy) during the first 10 months after elbow trauma showed postoperative recurrence after 46 months.

Cullen et al. [16] reported recurrence after early surgery (during the first months) for a small series of patients; they combined this with postoperative radiation therapy. Unfortunately, this series is heterogeneous as some of the patients underwent bone scan, and others did not.

Muheim et al. [17] recommended a series of bone scans for paraplegic patients in the case of heterotopic ossification, followed by a period of waiting for the phase of decreasing activity as the proper moment for operation. But the application of this recommendation in the case of synostosis is questionable.

Morrey and Harter offered this protocol: the resection of heterotopic ossification, avoiding articular cartilage damage; an atraumatic tissue handling; an accurate hemostasis; a meticulous lavage, with minimum bone dust production, avoiding neurologic injury; an accurately drain of the surgical field; and early postoperative motion [18].

**Figure 3:**
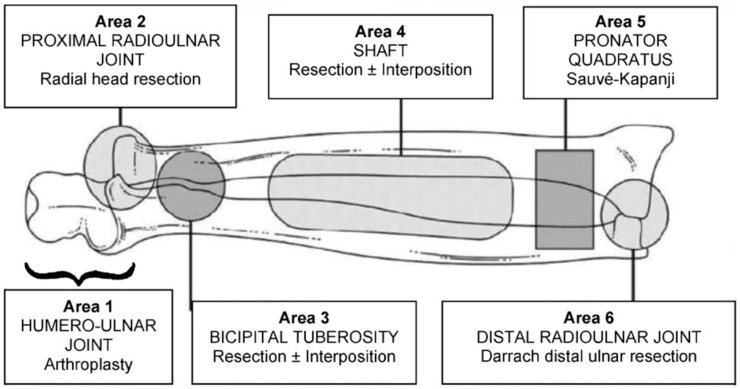
Hastings and Graham classification.

The surgical technique depends on the location of synostosis. Hasting and Graham summarized this in their classification (Figure 3).

For Type I, the Darrach procedure is recommended if the synostosis is localized in the distal radio-ulnar joint, and the Sauvé-Kapandji intervention is recommended if the synostosis is under the pronator quadratus.

For Type II and III A, the treatment is a simple resection of the synostosis with or without the interposition of soft tissue. In Type III B, a resection of the radial head is necessary [19], and in Type III C, an elbow arthroplasty.

The approaches depend on the site of the synostosis, its extension, and the severity of the articular surface and periarticular tissue injury. The posterolateral approach is recommended for Type IIIA synostoses (at or distal to the bicipital tuberosity) and for Type IIIB (at the level of the proximal radio-ulnar joint, or radial head) [4].

The posterior approach (with or without radial head excision) is indicated when the synostosis is extended with complete bone ankylosis of the joint, involving also the distal humerus (Type IIIC) [4].

The surgery is performed under general or locoregional anesthesia, with the patient in supine position with the arm on a hand table. A tourniquet is used to avoid excessive bleeding.

The posterolateral approach is made in Kocher’s interval, between the extensor carpi ulnaris and the anconeus muscles. During the surgery, the origins of these muscles are raised from the posterolateral edge of the proximal ulna, exposing the synostosis and the proximal third of the radius. When the ankylosis involves the posterior interosseous nerve, there is a risk of damaging the nerve during the resection. This is why it is recommended to place the forearm in pronation, as in this position the nerve lies more anteriorly and medially outside the operative field.

With the forearm in supination position, there is a high risk of injuring the nerve. In this case, the localization and isolation of the nerve should precede the resection of the synostosis [20]. The resection of the synostosis is made, protecting the soft tissue. The surface of the radius and ulna after debridement must be very smooth, without sharp edges, to avoid tissue injury. In patients with the synostosis extending to the proximal radio-ulnar joint, a proximal radial resection is recommended [19].

There are cases in which the bicipital tuberosity is caught in the synostosis. In this case, it is necessary to detach the bicipital tendon and, at the end of the procedure, to reattach it with anchors to the radial tuberosity.

The posterior approach is used when the synostosis is extended across the elbow [4]. This approach allows access to the lateral, posterior, medial, and anterior compartments. The radial nerve should always be isolated on the lateral side to avoid nerve injury. In many cases, the dissection requires elevation of the insertion of the brachioradialis, extensor carpi radialis longus, and brachialis muscle from the humerus and the releasing of the common extensor tendon insertion from the lateral epicondyle.

When heterotopic ossification is extended posteriorly, the dissection requires the elevation of the triceps so as to visualize the ossification with the posterior aspect of the humerus and the olecranon. Intraoperative fluoroscopy is necessary to check the quality of the resection after the excision. If the patient has posterolateral elbow instability, after the resection, the LCL must be reconstructed [21].

Sometimes, to restore full forearm rotation, resection of the proximal radius is necessary. A suction drain is applied at the end of the surgery to minimize residual hematoma, which can prevent bone formation.

Kelikian and Doumanian have had some success in two cases by developing a personal technique: they created a swivel to be inserted in the radial shaft, between the supinator and pronator teres muscle insertions completed with a muscle transfer (flexor carpi ulnaris or carpi radialis) and an ulnar styloid resection (for pain-free supination) [22].

## 4. Discussion

Whereas the surgical treatment is quite clear, there are some questions regarding the methods to be used to prevent recurrence. There is no consensus among surgeons, each having freedom to choose the preferred method, depending on their experience, training, or hospital capabilities.

(a) After synostosis resection, is interposition necessary or not?

The first question which should be debated in the treatment of Type II radio-ulnar synostosis is whether to use an interposition of soft tissue or not following the resection of the bone bridge. The idea underlying this technique is to prevent recurrence and to minimize scar formation.

There are many materials available: autograft, vascularized or not (fascia lata, adipofascial flaps, etc.), allograft (fascia, muscle), and synthetic materials (silicone, polyethylene, bone wax) [4,8,23,24,25,26,27,28]. The autograft material used for interposition has the advantages of being cheap and easy to procure. The fascia lata can be harvested from the same side as the one affected by synostosis during surgery. The disadvantage can be morbidity at the level of the donor site. An adipofascial flap is also efficient, but sometimes it is difficult to harvest (insufficient tissue) and is technically demanding (vascularized pedicle with adipofascial flap). An allograft tissue can be used (fascia lata), but it can trigger an immunological response with the resorbtion of the allograft.

To avoid these inconveniences, a number of synthetic materials have been developed (silicone, polyethylene, bone wax) with good results, but they are sometimes inaccessible and expensive.

These grafts are placed around the ulna, or the radius, and are secured with an absorbable suture. Usually, the case series offered by the authors are small and heterogenous, but the results suggest that interposition offers the best results. It is practically impossible to compare which method is the best because the number of cases is small and the results depend on the preference and surgical abilities of surgeons.

Jupiter and Ring interposed a free fat flap in a series of eight patients following synostosis resections; they left 10 cases without interposition. The results in both groups were functionally equivalent [4].

Yong-Hing and Tchang, Kawaguchi et al., and Muramatsu et al. had good results, treating a few cases with free vascularized fat transplant [28,29,30].

Failla et al. presented 12 cases of synostosis treated with interposition. Eight were treated with silicone gum leaf; two with muscle; one with fascia, fat, polyethylene, and silicone block. The results were excellent in four cases, the results were good in three cases, the results were moderate in four cases, and the results were poor in nine cases [8].

Bell and Benger treated three patients with vascularized anconeus muscle interposition with good results: mobility arcs of 100°, 110°, and 150° [25].

In 13 cases, Friedrich et al. used fascia lata (allograft to prevent donor site morbidity). After 30 months, nine patients had excellent results, two moderate, and two good [31].

A case of vascularized fat flap was presented by Sugimoto et al., who used it for the distal third of the forearm. The results after 1 year were 10° of pronation and 55° of supination [32].

In a series of seven patients, Sonderegger et al. reported a range of motion (ROM) of 70° of pronation and 70° of supination after using a vascularized adipofascial flap [33].

Full restoration of the ROM was obtained by Pfanner et al. in two cases treated with interposition with an allogenic fascia lata graft; no recurrence were observed after 2 years [14].

(b) Adjuvant therapy

To prevent heterotopic bone formation after surgery, over time, complementary methods have been described: treatment with non-steroidal anti-inflammatory drugs (NSAIDs) and low-dose radiation. In the case of the hip, these methods have shown some efficacy in the prevention of heterotopic bone formation; however, in the prevention of radioulnar synostosis recurrence, there are not enough data yet.

Bisphosphonates, theoretically, inhibit osteoid matrix calcification, but they have failed to show efficacy in preventing ossification after total hip replacement (THR) [34].

In multiple studies, Indomethacin has proven effective in preventing heterotopic ossification in the hip after THR. The daily dose was approximately 75 mg (25 mg three times a day) [35,36,37]. There is limited evidence to sustain this treatment in synostosis. Lytle et al. treated a patient with a dermal silicone sheet implant and Indomethacin. After surgery, he regained full pronation and near-normal supination, with no postoperative recurrence after 1 year. Pfanner et al. presented two cases treated with resection and fascia lata allograft with 2 months postoperative of Celebrex. The patients had a full ROM and no recurrence after 2 years [14].

There are studies that show, for animal fractures, that Indomethacin impairs fracture healing [38,39,40]. This makes Indomethacin less desirable for the prevention of ossification. Viola and Hanel have shown that there was no difference between patients who were administered the medication and others who were not in their series of patients [41].

In light of these observations, the administration of non-steroidal anti-inflammatory drugs can be used if a patient does not have other comorbidities (gastric or other digestive diseases, etc.). Usually, Indomethacin is used in doses of 25 mg, three times a day, with gastric protection, for 6 weeks.

After THR, low-dose radiation therapy has proved effective in preventing heterotopic hip ossification. Nevertheless, there are some studies also proving its effectiveness in preventing radio-ulnar synostosis. Abrams et al. reported two cases, one treated with a total of 1000 cGy divided over four daily doses and another treated with 700 cGy in one dose. Patients had no recurrence [42]. Cullen et al. noted no recurrence in a series of four patients treated with a single treatment of radiation of 800 cGy within 4 days of the resection [16]. T. Samartzis et al. voiced their concern regarding the risk of radiation-induced sarcoma [43]. Overall, the series involved was small and heterogenous, and this is why the use of low-dose radiation or Indomethacin is only recommended for the patients who do not have a risk of potential side effects (gastric ulcer or sarcomatous transformation).

(c) Postoperative rehabilitation

**Figure 4:**
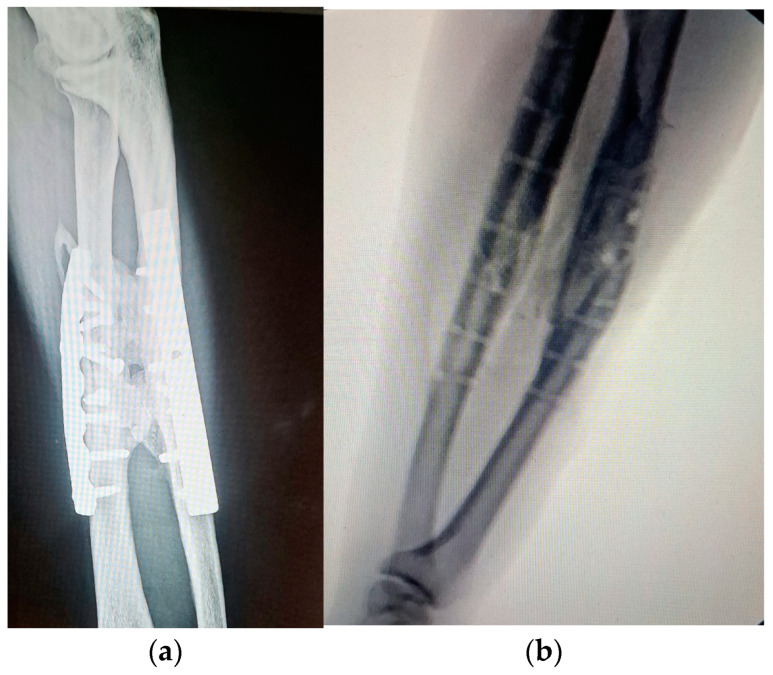
(**a**) A 49-year-old patient with fractures of both forearm bones and severe brain trauma who developed postoperative radio-ulnar synostosis. (**b**)The patient was operated on for synostosis a year after the first surgery. The patient received Indomethacin postoperatively for 6 weeks and began postoperative rehabilitation the next day.

**Figure 5:**
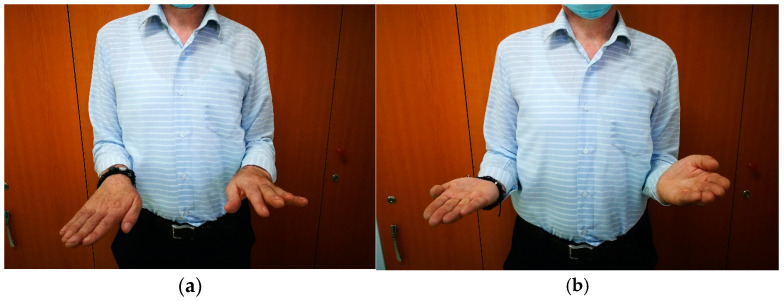
(**a**) Pronation and (**b**)supination movement after a few months from the surgical resection of the synostosis.

While there is general agreement regarding early intensive rehabilitation, there is no consensus about which is the best protocol. (Figure 4 and Figure 5). The protocols consist of passive and active physiotherapy sessions, splitting in maximum pronation and supination between them [31,44]. The rehabilitation time can sometimes be long and frustrating for the patient. Usually, after the surgery, mobility should be considerably improved. To maintain this result, early mobilization of the forearm (the next day after surgery in prone and supine position) is mandatory. To prevent stiffness, the patient can be placed with the forearm in extreme supination for a few hours, or in pronation position with the help of a cast or splint. The splints are alternately replaced. The patient should know that some degree of motion can be lost during the process due to the retraction of the soft tissue or insufficient mobilization, but if there is no recurrence, the results are very good.

(d) Recurrence rates

The risk of recurrence is between 6 and 35%, and a higher incidence has been noticed in patients with significant soft tissue injury and associated head (brain) trauma [2,4,8]. Recurrence is manifested by the progressive decrease in the range of motion. On X-ray, another bone formation was noticed. Likewise, the alkaline phosphate level increased in the blood tests. A possible reason for this complication is the lack of movement in part of the head of the injured patients.

## 5. Conclusions

Radio-ulnar synostosis is an uncommon complication which occurs in forearm trauma involving the interosseous membrane or after surgery involving the radius and ulna. Post-traumatic heterotopic ossification usually forms a proximal radio-ulnar synostosis in the forearm with secondary functional deficit (loss of pronation and supination of the forearm).

Prevention is preferable and involves the careful management of the fractures of the radius and cubitus to avoid the injury of the interosseous membrane as much as possible.

Surgical treatment is the gold standard and is determined by the Vince and Miller classification (adapted by Hasting and Graham) based on location. Screening radiography can be used to select the adequate time for the excision (resection is indicated when the bone margins appear mature on the radiographs).

Surgical intervention is recommended 6–12 months post-trauma, to allow for the bone maturation of the synostosis.

The surgical approaches are posterolateral and posterior. They depend on the site, extension of the ossification, periarticular tissue injury, and severity of the articular surface damages. 

After surgical resection, an interposition graft (using different materials) is used to prevent recurrence. Adjuvant therapy can be administered to patients with high-risk factors such as recurrence or traumatic brain injury. This consists of the administration of non-steroidal anti-inflammatory drugs (25 mg Indomethacin three times a day for six weeks) or low doses of radiation.

Postoperative rehabilitation is very important and should be intensive and early to maintain the postoperative ROM.

## 6. Future Directions

Because it is a very frustrating complication, detecting synostosis at an early stage is very important. A Mayo Clinic study classified ossifications based on their appearance, size, and functional implications, as follows: “hazy or granular immature” when the ossification is not very well formed; “mature limited” when the ossification is visible, but the area is small to moderate; “mature extensive” when the ossification is almost in contact with the cortical bone; and “bone bridge” when the ossification is connected with the cortical bone [45]. This protocol can be applied to all patients with severe forearm injury, especially for those with associated head trauma, to determine the size and the site of bone formation.

Based on the fact that bone formation is associated with a high level of alkaline phosphate, the periodic determination of this level in the blood during the first post-traumatic months can suggest the time of the occurrence of this complication.

After surgery, the prevention of recurrence can be performed by using interposition materials, such as an autograft or an allograft. Because the harvesting of fascia lata or fat vascularized tissue can sometimes be difficult and is associated with donor morbidity, the development of new synthetic materials, compatible with the human body, at affordable prices, is a direction for future development.

The discovery of the new anti-inflammatories with an anti-bone formation effect, besides the increase in the number of studies on the use of Indomethacin, can prove effective in synostosis recurrence.

Because the use of low doses of radiation has proven beneficial in some cases, it is expected that future studies will standardize and optimize this method.

The introduction of new physiotherapy methods, with an anti-inflammatory role, may influence the local evolution of the inflammation, thus preventing bone formation.

## Data Availability

The data presented in this study are available in references [1,2,3,4,5,6,7,8,9,10,11,12,13,14,15,16,17,18,19,20,21,22,23,24,25,26,27,28,29,30,31,32,33,34,35,36,37,38,39,40,41,42,43,44,45].
